# *Candida glabrata* Biofilms: How Far Have We Come?

**DOI:** 10.3390/jof3010011

**Published:** 2017-03-01

**Authors:** Célia F. Rodrigues, Maria Elisa Rodrigues, Sónia Silva, Mariana Henriques

**Affiliations:** CEB, Centre of Biological Engineering, LIBRO—Laboratório de Investigação em Biofilmes Rosário Oliveira, University of Minho, 4710-057 Braga, Portugal; elisarodrigues@deb.uminho.pt (M.E.R.); soniasilva@deb.uminho.pt (S.S.); mcrh@deb.uminho.pt (M.H.)

**Keywords:** *Candida* species, *Candida glabrata*, biofilm, candidiasis, resistance, antifungal, infection

## Abstract

Infections caused by *Candida* species have been increasing in the last decades and can result in local or systemic infections, with high morbidity and mortality. After *Candida albicans*, *Candida glabrata* is one of the most prevalent pathogenic fungi in humans. In addition to the high antifungal drugs resistance and inability to form hyphae or secret hydrolases, *C. glabrata* retain many virulence factors that contribute to its extreme aggressiveness and result in a low therapeutic response and serious recurrent candidiasis, particularly biofilm formation ability. For their extraordinary organization, especially regarding the complex structure of the matrix, biofilms are very resistant to antifungal treatments. Thus, new approaches to the treatment of *C. glabrata*’s biofilms are emerging. In this article, the knowledge available on *C. glabrata*’s resistance will be highlighted, with a special focus on biofilms, as well as new therapeutic alternatives to control them.

## 1. Introduction

### 1.1. Biology of Candida glabrata

**Figure 1:**
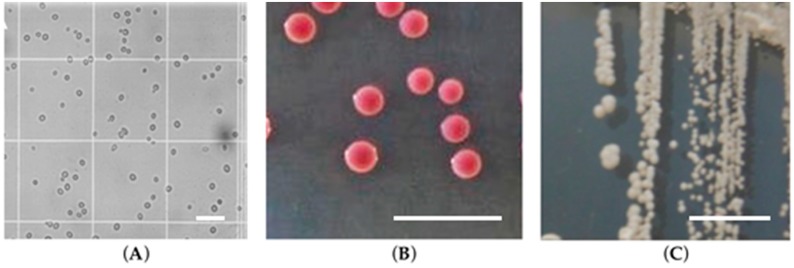
*Candida glabrata* cells: (**A**) microscopy structure; (**B**) on CHROMagar^TM^ *Candida*; (**C**) on Sabouraud dextrose agar (adapted from [4]). The scale corresponds to 50 μm with a magnification of 200×.

Historically, *Candida glabrata* strains were originally classified in the genus *Torulopsis* due to its lack of filaments forms formation. However, in 1978 it was determined that the ability to form hyphae and/or pseudohyphae was not a reliable distinguishing factor of members of genus *Candida* species, and it was proposed that *T. glabrata* should be classified in the genus *Candida*, due to its human pathogenicity [1]. In fact, in contrast to other *Candida* species, *C. glabrata* is not polymorphic, growing only as blastoconidia and regarding the genetic aspects of *Candida* species, a critical distinguishing characteristic of this species is its haploid genome, in opposition to the diploid genome of *Candida albicans* and other *Candida* species. It should be highlighted that *C. glabrata* cells (1–4 µm) are noticeably smaller than *C. albicans* (4–6 µm), *Candida tropicalis* (4–8 µm), and other *Candida* species blastoconidia [2] (Figure 1). In Sabouraud dextrose agar culture medium, *C. glabrata* strains forms glistening, smooth, and cream-coloured colonies, which are relatively indistinguishable from those of other *Candida* species except for their relative size (Figure 1), which can be quite small [3]. On CHROMagar^TM^ *Candida* (CHROMagar, Paris, France), a differential agar medium, it is possible to distinguish a number of different *Candida* species by colour; as a result of distinct biochemical reactions, *C. glabrata* colonies appear white, pink to purple (Figure 1), in contrast to *C. albicans* colonies which are blue-green. Concerning the biochemical reactions of *Candida* species, *C. glabrata* ferments and assimilates only glucose and trehalose, contrary to *C. albicans*, which ferments and/or assimilates a high number of sugars excluding sucrose [4,5]. Whereas *C. albicans, Candida parapsilosis*, and *C. tropicalis* are moderately closely related species of the CUG clade, which share a unique codon exchange from leucine to serine, *C. glabrata* is actually a “misnomer”, for it is really much more closely related to the *Saccharomyces cerevisiae* than to *C. albicans* [6,7]. As mentioned, oppositely to the other *Candida* species, but equally to its cousin *S. cerevisiae*, *C. glabrata* is strictly haploid and typically grows only in the yeast form [8].

### 1.2. Epidemiology and Virulence Factors of Candida glabrata

For many years *C. glabrata* was considered a relatively non-pathogenic saprophyte of the normal flora of healthy individuals and certainly not readily associated with serious infections in humans. However, following the widespread and increased use of immunosuppressive therapy together with broad–spectrum antibiotic and antifungal therapies, the frequency of mucosal and systemic infections caused by *C. glabrata* has been growing significantly [9]. In fact, whilst mycological studies have been shown that *C. albicans* represents approximately 80% of the clinical isolates, in the last few decades, the number of candidiasis due to non-*Candida albicans Candida* (NCAC) species has meaningfully raised, namely in regard to *C. glabrata* strains [1,10,11]. Some studies suggest that fungemia has been associated with NCAC species [11,12,13]. The incidence of *C. glabrata* is higher in adults than in children, and is lower in neonates [14,15]. In the European Confederation of Medical Mycology survey, the frequency rates of candidiasis attributed to *C. glabrata* were around 14% [16] and 15% of all *Candida*-related systemic bloodstream infections [17,18]. This is extremely important since, compared to other *Candida* species infections, the mortality rate associated with *C. glabrata* is the highest [19].

Subsequently to the introduction of the highly active antiretroviral therapy, a reduction in the percentage of oropharyngeal infections, the colonisation by *Candida* species, and a decline in the frequency of fluconazole resistance in patients with HIV infection have been recorded [20]. However, *Candida* species are still the most frequent cause of systemic mycosis in our time [21,22]. During 1995–1996 and 1997–1998, a national programme of surveillance of bloodstream infections in the USA [23], and the SENTRY international programme of surveillance of bloodstream infections in the USA, Canada, and South America [24], showed the rising importance of NCAC, which accounted for between 44% and 48% of cases of fungaemia. Among NCAC, *C. glabrata* clearly stood out, with an increased prevalence observed through the study period in all three geographical regions, becoming the second most frequent species after *C. albicans* in the USA and Canada [23,24] and with a mortality rate associated with bloodstream infections of 49% in a retrospective series of 139 cases [25]. In the European SENTRY programme *C. glabrata* was the third most common NCAC, after *C. parapsilosis* [26]. In considering the SENTRY programme of USA from 1997–1999, NCAC were usually more susceptible to fluconazole, but continued surveillance is needed to confirm this tendency, as it is known that this may be not accurate in present days, mostly for *C. glabrata* and *Candida krusei*, which are known to be intrinsically resistant to fluconazole [23,24,26,27,28,29,30,31].

Until recently, few studies had evaluated independent risk factors associated with nosocomial *C. glabrata* acquisition and subsequent infection. Little is known about the hospital reservoirs of *C. glabrata*, but with *C. albicans*, probable sources include a complex interaction of environmental and human reservoirs [32]. Vasquez and colleagues [33] revealed that patients with a new acquisition of *C. glabrata* had extended and repeated hospitalizations prior to antifungal use compared to patients with no *Candida* species exposition. *Candida glabrata* has been also often isolated from patients with oral candidiasis, alone or coupled with *C. albicans* clinical isolates [4,34] and thus, has been related to recurrent systemic infections [35,36]. The propensity of *C. glabrata* for dissemination and the high mortality associated could be related to the virulence factors that this species exhibits, namely the elevated rates of resistance to the traditional antifungals.

The relatively nonpathogenic nature of *C. glabrata* in animal models [37,38] suggests that it has only few virulence attributes. However, the high mortality rate and the rapidity of the spread of disease would suggest the contrary [1]. In fact, in opposition to its inability to form hyphae and/or pseudohyphae and secret proteases, *C. glabrata* retain many virulence factors such as the capacity to secrete phospholipases, lipases, and haemolysins that contribute towards an extreme aggressiveness resulting in a low therapeutic response and serious recurrent candidiasis [10,39]. However, its most worrying virulence factor is its strong capability to form biofilms [10,40,41]. Biofilms are known as surface-associated communities of microorganisms embedded in an extracellular matrix [42,43], which confer significant antifungal therapy and host immune responses [4,10,41]. *Candida glabrata* clinical isolates have the ability to form a compact biofilm structure in different multilayers [40,41], with proteins, carbohydrates (e.g., β-1,3 glucans), and ergosterol in their matrixes [40,41,44]. The first step of *C. glabrata* biofilm development is adhesion and/or colonisation of yeast cells to an abiotic or/and biotic surface [4,10]. Adhesion is an extremely important step, not only in the biofilm formation, but also in the infections processes, and the extent of adhesion is dependent on *C. glabrata* cells’ characteristics, and host and/or abiotic surface properties, such as cell-surface hydrophobicity and cell wall composition [4,45]. The *Candida glabrata* cell wall is the site for physicochemical interactions between the microorganism and the surfaces, leading to its adherence. Despite the lack of studies concerning this issue, it is assumed that the cell surface of *C. glabrata* cells reportedly exhibit a degree of hydrophobicity comparable with *C. albicans* [46]. Interestingly, however, while the hydrophobicity of *C. albicans* was extremely sensitive to specific growth conditions, numerous isolates of *C. glabrata* were relatively insensitive to those same growth conditions [47]. Similar to *C. albicans*, *C. glabrata* adhesion phenomenon is mediated by epithelial adhesins (*Epa*) that have a comparable structure to the Als proteins [48]. The family of *EPA* genes are composed of 17–23 genes depending on the strain, however, *EPA1*, *EPA6*, and *EPA7* are the most important adhesins [49]. Deletion of the *EPA1* gene reduces *C. glabrata* adherence in vitro to host epithelial cells [50] and the adherence of this adhesin was inhibited in the presence of lactose [51]. In addition, usually *C. glabrata* strains are unable to express *EPA6* in vitro, however, it is expressed during urinary infection, due to low levels of nicotinic acid [51]. Groot et al. [52] identified another family of adhesins involved in the first stage of *C. glabrata* biofilm development, namely Awp adhesins. Initially, four Awp adhesins (Awp1–4) were identified using liquid chromatography tandem mass spectrometry [52], and a subsequent study revealed the gene expression profile of the seven Awp adhesins (Awp1–7) [53]. Initial attachment of *C. glabrata* cells is followed by cell division, and this proliferation leads to the formation of a basal layer of anchoring microcolonies, with subsequent biofilm maturation [4,44]. Biofilm conditions and high cell density are adhesion inducers, activating *EPA6*, whereas *EPA1* is triggered typically in the lag phase and the *C. glabrata* biofilm maturation is characterized by the production of the extracellular matrix [4,54]. A study using isolated mutant strains allowed the identification of four other genes involved in biofilm formation: silent information regulator (*SIR4*), telomere-binding (*RIF1*), *EPA6*, and serine-threonine protein kinase, *YAK1*.

## 2. General Mechanisms of Antifungal Drug Resistance

Clinical resistance is the result of a failure in the infection treatment [55]. Regarding susceptibility or resistance, *Candida* is defined as susceptible or resistant by the level of antifungal drug activity associated with a high likelihood of therapeutic success or therapeutic failure, correspondingly. There has been an epidemiological change from *C. albicans* to NCAC species, with *C. glabrata* and *C. krusei* emerging as important and potentially antifungal resistant causes of candidaemia [56]. It is important to mention that *C. glabrata* has been frequently reported as exhibiting variable karyotypes between isolates [57,58,59,60,61,62,63,64], and several studies with *Candida* species have demonstrated that these karyotypes are relatively stable, suggesting that the karyotype of virulent species is more stable than virulent ones [65]. The major karyotypic differences between *C. glabrata* strains are linked to a small number of chromosomal translocations. Along with variation in the subtelomeric *EPA* genes, the other genomic rearrangements are copy number variations in tandem gene repeats, encoding putative, or known cell wall proteins [58].

Bader et al. [66] analysed the derivates in *C. glabrata* strains’ genome, which were shown to be indistinguishable by multi locus sequence typing, but dissimilar phenotypic groups that were linked with specific karyotypic changes were also spotted. Chromosomal aberrations and functional adaptations can occur during infection and under antimicrobial therapy, but also under laboratory conditions deprived of extreme selective pressures, and can significantly affect phenotypic properties (e.g., the cell wall carbohydrate composition and quantitative changes in adhesion genes expression), being noticed slightly less than subtelomeric genes loss or differences in the number of macrosatellite repeats within adhesion genes. Another study also revealed that chromatin alterations could happen as essential strategies of survival, which would simplify a reprogramming of cellular energy metabolism in macrophage-internalized *C. glabrata* cells, and provide protection against DNA damage [67]. Thus, similar to all *Candida* species, *C. glabrata* have the competence to respond to environmental alterations, allowing them to adapt to the presence of antifungal agents, thereby providing protection against antimicrobial therapies [68].

Cell wall fluctuations, but mostly its immunoevasion and intracellular persistence, may be the crucial factors in the great ability of *C. glabrata* to persist in the course of multiple antifungal treatments and to develop multidrug resistance [69,70]. Consequently, different mechanisms of resistance vary among drugs, typically because of the mode of action of each class of antifungal. There are, presently, three main antifungal classes: azoles, polyenes and echinocandins. The azoles are known to have fungistatic activity, targeting the ergosterol biosynthetic pathway, by binding to the Cyp51 family of cytochrome P450—the 14-α sterol demethylases that are encoded by *ERG11*. They are responsible for the lack in the capacity to build and renew sterols in the cellular membranes, changing membrane fluidity and function of vital processes such as signalling, transport, exocytosis, and endocytosis [10]. Fluconazole, voriconazole, itraconazole, clotrimazole, and posaconazole are main examples. The polyenes (e.g., amphotericin B, nystatin) and echinocandins (e.g., micafungin, caspofungin, anidulafungin) are fungicidal drugs. The first ones bind to the ergosterol of the fungal cell membrane establishing transmembrane aggregates pores, which causes membrane depolarization which subsequently increases its permeability to monovalent protons and cations. This allows the passage of intracellular molecules to the external environment, initiating an osmotic imbalance, and finally cell death [71,72,73]. Echinocandins interfere with the fungal cell wall synthesis through a non-competitive inhibition of β-1,3-glucan synthesis [74], which results in the weakening of the cell wall, breakdown of cellular integrity and, finally, cell lysis [75].

### 2.1. Azole’s Resistance

During the past few decades there was a remarkable increase in mucosal infections caused by *Candida* species due to the increase of immunosuppressive diseases (e.g., cancer, AIDS), which was associated with an extraordinary emergence of resistance to azoles [76]. In the early 1990s, fluconazole became the first choice drug in the treatment and prophylaxis of oro-oesophageal candidiasis, and resistance was subsequently described in up to 41% of the patients in the following years [77,78,79]. Also, an increase was observed in the cases of fungaemia caused by NCAC species, specially *C. glabrata* and *C. krusei* [19,80,81,82]. As recognized, *C. glabrata* grows only as a yeast form in vivo and its adhesion is relatively weak [83,84,85]. Thus, it is believed that the increase of *C. glabrata* infections is due to that same inherent low susceptibility to azoles [86] and that the acquired resistance is a result of rare mutations that are selected by drug pressure [87]. Studies appear to conclude that the azole resistance develops gradually as a consequence of successive adjustments due to the continuous pressure exerted by the drug [87,88,89]. The acquired resistance of *C. glabrata* to azole drugs is linked to several mechanisms, but the most common is the induction of efflux pumps, encoded by the *ABC*-transporter genes (*CDR1* and *CDR2*, *SNQ2*) or to *MDR* belonging to the major facilitator superfamily (MFS) that lead to decreased drug concentration [90]. In *C. glabrata*, the transcription factor Pdr1 is involved in resistance to azoles through upregulation of *CDR1*, *CDR2*, and *SNQ2* [91]. The mitochondrial dysfunction associated with the development of the “petite mutants”, which have mitochondrial DNA deficiency and upregulate the *ABC* transporter genes, highly amplifying resistance to azoles, leading to a drastic improvement of fitness in *C. glabrata*. These mutants upregulate *ABC* transporter genes, displaying enlarged resistance to azole drugs [92]. A number of *ABC* transporters, including Cdr1, Pdh1 (also known as *CDR2*), Yor1, and Snq2, contribute to xenobiotic drug efflux. The transcription factor Pdr1 is the main regulator of *ABC* transporter gene expression and the key component of Pleiotropic Drug Resistance (*PDR*) [93,94]. In *C. glabrata*, Pdr1 forms a heterodimer with Stb5 in *S. cerevisiae* and transcriptional analysis pointed out a shared region among the homologues of these two genes, *PDR1* and *STB5*, and many of the genes upregulated by overexpression of *PDR1* were upregulated by the deletion of *STB5.* Accordingly, the *PDR1* overexpression and *STB5* deletion are correlated [95,96]. It was found that the overexpression of *STB5* in *C. glabrata* represses azole resistance, while its deletion produces a minor intensification in resistance. Expression analysis assays recognized that *STB5* shares many transcriptional targets with *PDR1* but, unlike the second, it is a negative regulator of pleiotropic drug resistance (including the *ABC* transporter genes *CDR1*, *PDH1*, and *YOR1*) [91,96]. A study by Farahyar et al. [97] demonstrated that *CDR1* and *CDR2* genes are expectedly upregulated in azole-resistant isolates (≥2-fold) and that, fatty acid activator 1 (*FAA1*) gene presented a ≥2-fold expression in resistant isolates, when compared to the susceptible isolates and the reference strain. The work also revealed that not only the *ABC* transporter genes, but also small hydrophobic compounds and lipid metabolism may have a huge responsibility in azole drug resistance of *C. glabrata* [97]. A study of Ferrari and colleagues [91] involving transcription profiling with microarrays showed that more than 385 genes are differentially regulated by a selected number of the gain-of-function mutations (GOFs) expressed in the same genetic background, with a minimal overlap in co-regulated genes. *CDR1* and *PUP1* (for *PDR1* upregulated and encoding a mitochondrial protein) were generally upregulated by all tested GOFs. While both genes mediated azole resistance, their deletions resulted in a decline in virulence and a decrease in tissue load. Their individual overexpression was shown to partially restore phenotypes obtained in clinical isolates [91]. Kaur and colleagues [98] made a screening of a library of 9216 random insertion mutants, and identified a set of 27 genes, which upon mutation, conferred alterations in fluconazole susceptibility in *C. glabrata*. These genes included *ABC* transporters (*PDR5* and *PDR16*), genes involved in retrograde signalling from mitochondria to nucleus (*RTG2*) and genes involved in diverse cellular functions (activation of RNA polymerase II transcription, calcium homeostasis, ribosomal biogenesis, mitochondrial function, nuclear ubiquitin ligase function, and cell wall biosynthesis). Similarly, using a mutant defective in calcium uptake, the same authors noticed that the strains with a flaw in a putative plasma membrane calcium channel were modestly more susceptible to fluconazole, but revealed a significant loss of viability upon prolonged fluconazole exposure. This result suggests that calcium signalling is necessary for the survival of azole stress in *C. glabrata* and that, in the absence of Ca^2+^ signalling, fluconazole has a fungicidal rather than a fungistatic effect on *C. glabrata* [99]. Another azole-related resistance mechanism is the decreased affinity, or even incapacity, of these drugs to bind. The high ability to upregulate *ERG11*, *CDR1*, and *PDR1* expression is, normally, followed by azole exposure [100]. All the genes linked to the biosynthesis of ergosterol are likely to be upregulated in the case of azole pressure, nonetheless, *ERG 1*, *3*, *6*, *7*, *9*, and especially *ERG11*, are the most studied *ERG* genes. *ERG11*, which converts lanosterol into 4, 4-dimethylcolesta-8,14,24-trienol, is markedly more mentioned as a central point regarding the increase of ergosterol bioproduction, in response to the azole attack on the *C. glabrata* cell membrane [87,101]. Potential mechanisms for the azole resistance include a small affinity of its lanosterol 14α-demethylase. The resistance mechanisms in *ERG11* occur through the acquisition of point mutations in the gene encoding for the Erg11. Quite a few mutations in *ERG11* have been described, but only a few are directly connected to azole resistance [102,103]. The overexpression or upregulation of the target enzyme of the azoles is also an important resistant mechanism linked to azole drugs [55]. Recently, a study in three different hospitals in Poland was developed in order to determine the mechanisms of resistance to azoles in *C. glabrata* clinical isolates in this country [104]. The authors used a Sensititre Yeast One test and discovered that, from the 81 studied strains, 18 were resistant to fluconazole, and 15 were cross-resistant to all other azoles tested. RT-qPCR studies showed that 13 of 15 azole-resistant strains presented upregulation of the *CDR1* gene encoding the efflux pump, but no upregulation of the expression of *CDR2*. Also, no upregulation of the *ERG11* gene was observed. This study confirms that the gene profile of the resistant isolates of *C. glabrata* azoles is variable between countries and strains, although certain genes are commonly up or downregulated [104]. Miyazaki et al. [99] studied the effects of calcineurin, a serine-threonine-specific protein phosphatase [105]. This protein emerged as a new target of antifungal therapy founded on studies in several pathogenic fungi, probably because azole antifungals and calcineurin inhibitors have mild synergistic effects against *C. glabrata* wild-type strains [98,106,107]. The results of this group have shown that the *C. glabrata* calcineurin mutant presented augmented susceptibility to azoles and cell wall-damaging agents and had lower virulence. Though the mutant lacking Crz1 presented a cell wall-associated phenotype intermediate to that of the calcineurin mutant and was modestly reduced in virulence, it did not improve azole susceptibility, thereby suggesting that calcineurin regulates both Crz1-dependent and independent pathways depending on the type of stress [99]. Chen et al. [108] disclosed that *AP1* (which encodes a transcription factor related to stress responses) plays a critical role in reaction to various stresses in *C. glabrata* and decreases the stress through transcriptional activation of its target genes, including *FLR1*. The deletion of this gene only caused an amplified sensitivity to fluconazole. *Candida glabrata* clinical isolates are known to have the aldo-keto-reductase superfamily upregulated in the resistant isolates. RT-qPCR analysis revealed a *AKR* mRNA expression twice of that seen in the sensitive isolates, associated with increased fluconazole and itraconazole resistance, thus suggesting that upregulation of the *AKR* gene might give a new insight into the mechanism of azole resistance [49,109]. Although isolation of such *C. glabrata* mutants from patients has been rarely reported, Ferrari et al. [92] have successfully characterized two sequential and related *C. glabrata* isolates recovered from the same patient undergoing azole therapy: BPY40 (azole susceptible) and BPY41 (azole resistant). BPY41 had a mitochondrial dysfunction with upregulation of the *ABC*-transporter genes of *C. glabrata*. Testing the virulence of the “petite mutants” in mice with systemic and vaginal murine infection models, the authors showed that, even with in vitro growth deficiency, BPY41 was more virulent than BPY40. The authors also found an increase in the oxido-reductive metabolism and in the stress response in BPY41, which was consistent with mitochondrial dysfunction, and that certain genes involved in cell wall adaptation were upregulated in BPY41 compared to BPY40 [92]. Finally, Taff et al. [110] identified two glucan transferases and an exo-glucanase that deliver glucan from the cell to the extracellular matrix, playing a biofilm-specific role, by mediating the distribution and organization of mature biofilm matrix.

### 2.2. Polyene’s Resistance

The first cases of resistance to amphotericin B arose in parallel with the increase in the number of invasive infections caused by several genus of fungi, many of them with primary or intrinsic resistance to amphotericin B and usually associated with a high mortality [111,112,113]. Although *C. glabrata* is frequently considered to be susceptible to amphotericin B, it has a clear tendency to have higher minimum inhibitory concentration (MIC) values to polyenes than *C. albicans* [76,114]. Polyene resistance is still poorly understood and not well documented, particularly in *C. glabrata*, but the molecular mechanisms primarily include the replacement of some or all of the polyene-binding sterols, and the reorientation or the camouflaging of studying a clinical isolate of *C. glabrata*. Vandeputte and colleagues [115] revealed lower ergosterol content in its membrane in comparison to the wild type, and also found a nonsense mutation in the *ERG6* gene that leads to a decrease in ergosterol content. Discrepancies of the cell wall were also observed, which were associated with developed susceptibility to cell wall-perturbing agents, with a high rate of cell mortality [115]. In another clinical isolate of *C. glabrata* recovered from a patient treated with amphotericin B and with a poor susceptibility to polyenes, a deficiency of ergosterol and an accumulation of late sterol intermediates was detected, emphasizing a defect in the final steps of the ergosterol pathway. Sequencing exposed a unique missense mutation in *ERG6* (substitution of a cysteine by a phenylalanine in the corresponding protein). RT-qPCR demonstrated an overexpression of the genes that encode enzymes involved in late steps of the ergosterol pathway. The complementation of this strain with a wild-type copy of the *ERG6* gene regenerated the susceptibility to polyenes and the standard morphology [116].

### 2.3. Echinocandin’s Resistance

Echinocandins are the first-line agents in the treatment of candidaemia [74]. Three mechanisms can induce the reduced echinocandin susceptibility [55]: acquired *FKS* mutations [117] which confer low β-1,3-d-glucan synthase sensitivity, higher MIC values, and clinical failure [118]; adaptive stress responses, which result in high cell wall chitin content with a paradoxical growth in vitro [119]; and finally, intrinsic *FKS* mutations, also resulting in elevated MIC levels (but in a lower level of reduced β-1,3-d-glucan synthase sensitivity when compared with the acquired *FKS* mutations) [118,120,121]. The *GAS* gene family is also a regulator in the production of β-1,3 glucan in this species [52]. *GAS1*, *GAS2*, and *GAS5* are glycosylphosphatidylinositol (GPI)-anchored cell surface proteins [122], which are involved in the production of β-1,3 glucan in *C. glabrata* [123]. A study performed from patients with *C. glabrata* bloodstream infections showed that the resistance to echinocandins increased from 4.9% to 12.3% between 2001 and 2010. In addition, among the 78 fluconazole resistant isolates, 14.1% were resistant to one or more echinocandin and almost 8% of the isolates had a *FKS* mutation (*FKS1/FKS2* mutations), which appeared due to a prior echinocandin therapy. Additionally, nearly all revealed intermediate or resistant MIC values to one echinocandin [124]. Thompson III et al. [125] performed sequentiation of hot spots studies in 2008, known to confer echinocandin resistance, and the fallouts revealed an F659V substitution within the *FKS2* region of the glucan synthase complex [125]. Curiously, micafungin MIC levels of *C. glabrata FKS* hot spot mutant isolates were observed to be less elevated than those obtained for the other echinocandins, showing that the efficacy of micafungin could be differentially dependent on specific *FKS* gene mutations [126]. Shields et al. [117] analysed several echinocandin MIC levels and found that the average MICs values of caspofungin and anidulafungin were higher for patients who failed therapy. Several *Candida* species isolates observed in vitro reflect a curious high-dose paradox which is being linked to a complex network of pathways, causing slightly elevated MIC levels, in which cells appear to regain susceptibility at high levels of a drug [127,128]. This has the potential to contribute to clinical resistance [55] and it is extremely important to differentiate these low-level drug tolerance and adaptive mechanisms from the Fks1-mediated mechanisms that have been observed in clinical isolates and can result in treatment failure [55,129].

## 3. Resistance Mechanisms Related to Biofilms

Fungi in general, and *Candida* species in particular, are not motile microorganisms. The biofilm structure, thus, reflects the sequence of cell division events that occur during a biofilm development and results in an exceptionally resistant profile of the biofilm cells to one or several antifungal drugs. The infections are complicated by the presence of robust inducible gene networks encoding different proteins that confer tolerance or resistances to many available antifungal drugs [130]. These resistances can be classified as either microbiological or clinical [55]. The first is defined by the presence of a developed or mutational resistance mechanism to a specific drug. It depends directly on the microorganism and it is distributed into two groups: primary or innate, in which fungi are resistant prior to drug exposure, and secondary or acquired, when it appears in response to a drug exposure.

Several general mechanisms of biofilm drug resistance are thought to confer resistance to multiple classes of antifungals in *Candida* species biofilms: the up-regulation of efflux pumps, cell wall composition, increased cell density and *quorum sensing* effect, presence of an extracellular matrix, changes in metabolism, the presence of persister cells, and cellular signalling and stress responses [101,130,131,132,133].

### 3.1. Up-Regulation of Efflux Pumps and Cell Wall Composition

Two main classes of efflux pumps contribute to antifungal drug resistance: the ATP binding cassette (*ABC*) transporter superfamily containing *CDR1* and *CDR2*, and the MFS superfamily containing *MDR1* [130,134,135]. While under treatment with antifungals, biofilm cells up-regulate these transporters within six hours of surface contact both in vitro and in vivo, even in the absence of drug [136,137,138,139]. In 2013, our group [41] described that the usual *ABC* transporters were upregulated in biofilms of three *C. glabrata* strains. Similarly, another *ABC* transporter, *PDR1*, was evaluated and was found to be overexpressed. These alterations were linked to modifications in the structure of *C. glabrata* biofilms by creating cell clusters, which could be a possible mechanism of biofilm tolerance to fluconazole. The surface adherence alone seems enough to intensify the expression of the genes encoding the efflux pumps [140], which are also up-regulated in mature biofilms, demonstrating that they continue to mediate drug resistance throughout biofilm development [141,142,143].

### 3.2. Increased Cell Density and Quorum Sensing

It is well recognized that the inoculum size can affect susceptibility results [130,144,145,146,147]. Thus, using the microtiter method to test *Candida* species for drug susceptibility, an optimal inoculum size range was defined as a clinical standard. Indeed, if this cell concentration is augmented in the drug resistance assays, resistance specifically to fluconazole, ketoconazole, amphotericin B, and caspofungin is increased up to twenty-fold [147]. On the contrary, if biofilms are dissociated and analysed at a lower density in the same assays, they show drug susceptibilities at the level of planktonic cells evaluated at the same cellular density.

In the biofilm environment, *Candida* species cells have the ability to communicate with each other via *quorum sensing* (via numerous signalling molecules), which is directly dependent on the cell density [148].

### 3.3. Extracellular Matrix

In *Candida* species biofilms, carbohydrates, proteins, and nucleic acids form the extracellular matrix that surround the cells in the biofilms [149,150,151,152,153,154]. It is thought that biofilms prevent, to a greater or lesser extent, the penetration of antifungal drugs through their structure, by the establishment of a diffusion barrier, which acts as an ion-exchange resin, binding charged antibiotic molecules, contributing to biofilm drug resistance, including in the case of *C. glabrata* [44,150,155]. The most important components are the β-glucans, which are polymers of the fungal cell wall and are a substantial constituent of the biofilm *Candida* species matrix. When induced, the disruption of β-1,3-glucans or a β-1,3-glucanase treatment have been shown to increase susceptibility of biofilms to fluconazole and the addition of exogenous β-1,3 glucans has been demonstrated to result in the rise of resistance to fluconazole in planktonic cells [155]. Additionally, it is possible that biofilms can also sequester amphotericin B, as it has been shown that β-1,3-glucans can bind specifically to this drug [44,156]. It is known that planktonic cells generally rely on irreversible genetic changes to maintain a resistant phenotype, while biofilms are able to persist due to their physical presence and the density of the population, which affords an almost inducible resistant phenotype notwithstanding of distinct genetic alterations [130,157]. The application of DNA microarray and proteomic technologies can facilitate a more detailed analysis of the biofilm lifestyle [158,159] Specific biofilm formation genes are being brought up regarding different roles in biofilm resistance: peroxisomal catalase (*CTA1*), the biosynthesis and degradation of tyrosine genes (*ARO*), the muscle creatine kinase (*MSK*), the heat shock protein 90 (*HSP 90*), the sphingolipid biosynthesis (*SKN 1* and *KRE1*), *SIR*, *RIF*, and, finally, the extracellular matrix (ECM) regulators: zinc regulated *genes (ZAP1)*, g-carbonic anhydrase (*GCAL1*), alcohol dehydrogenase (*ADH5*), and also cell surface hydrophobicity (*CSH1*) [130,159].

### 3.4. Metabolism and Stress Response

Alterations in temperature or specific nutrients, restricted nutrient availability, ionic stress, and variations in osmolality and oxidative stress are all acknowledged as antifungal resistance mechanisms of biofilms. A study showed that the resistance to chlorhexidine, fluconazole, amphotericin B, and nystatin increased as biofilms mature over time, corresponding to a growth in metabolic activity over biofilm maturation. However, in these experiments, cell number was not controlled, thus making it unclear if this is a true demonstration of metabolic activity [159]. Baillie and Douglas [160] have shown that, at lower growth rates, planktonic cells are more resistant to amphotericin B, and biofilms are equally resistant over a range of growth rates, thereby suggesting that growth rate plays only a minor role in *Candida* species biofilm drug resistance. In another study [161], the same authors demonstrated that neither glucose nor iron limitation disturbs *Candida* species biofilm resistance to amphotericin B. However, iron limitation increased the susceptibility of dispersed daughter cells from biofilms to amphotericin B, detected by a number of cells which induce responses by signalling pathways [162]. Seneviratne et al. [163] showed that there is a positive regulatory protein response associated with the stress response in biofilms of stressed *C. glabrata*, as displayed by the heat shock and other stress proteins (Hsp12, Trx1, and Pep4).

### 3.5. Persister Cells

Inside the biofilm cell population, persister cells form a unique group that is formed randomly, phenotypically dormant, highly tolerant to antifungal drugs [164], and which is a key mechanism of resistance in chronic infections [130]. Yeast persister cells were first discovered as a small population in *C. albicans* biofilms [165,166]. These cells were extremely drug resistant in a manner that was independent of drug efflux pumps and the composition of the cell membrane. Al-Dhaheri and Douglas [166] reported persister cells in biofilms treated with amphotericin B from isolates of *Candida* species. *Candida* species persister cells are exclusively recovered from biofilms and not from planktonic populations, notwithstanding their growth phase, and involve attachment to a substrate to initiate the dormant phenotype. These cells are believed to be a phenotypic variant of the wild type strain, for they are the result of a biofilm with new subpopulations [133]. Bojsen and colleagues [167] performed a study in order to evaluate whether resistance mechanisms on amphotericin B were shared between biofilm and planktonic populations. A multiplexed barcode sequencing screening of a combined group of gene-deletion mutants cultivated as biofilm and planktonic cells associated with an assay for resistance to the ergosterol-targeting fungicide amphotericin B was executed. The results revealed that the biofilm and planktonic population had substantial overlap in amphotericin B-persistent mutants. Also, the authors were able to demonstrate that the mutants defective in sterol metabolism, ribosome biosynthesis, and in the *TORC1* (ubiquitin binding activity, role in cellular response to starvation, regulation of cell growth, etc.) and in the Ras pathways (protein signal transduction) displayed an amplified persistence when treated with amphotericin B. The *ras1*, *ras2*, and *tor1* mutants had a high-persister phenotype compared to wild-type biofilm and planktonic cells exposed to the *TORC1* pathway inhibitor rapamycin, and, on the other hand, the inhibition of *TORC1* with rapamycin similarly improved the proportion of persisters in *C. glabrata.* With these results, the authors demonstrated that a decreased *TORC1*-mediated induction of ribosome biosynthesis via Ras can originate the development of amphotericin B-persister cells in planktonic populations, but also in biofilms [167].

## 4. Cross and/or Multidrug Resistance

Antifungal drug resistance is particularly more serious when it develops not only against the administered drug, but also to other non-related chemical compounds, and *C. glabrata* has emerged as a major health threat since it also rapidly acquires resistance to multiple drug classes. The use of echinocandins and development of cross-resistance is creating apprehension, for the signs of multidrug resistance amongst azoles and echinocandins have already been described [168,169,170]. Studies with clinical isolates obtained from patients in several epidemiological studies show that not only has multiple antifungal resistance been described in isolated events, but also that cross-resistance among more than one class of antifungal drugs is growing [171]. As a result, 11.1%–58.3% of *C. glabrata* isolates resistant to echinocandins had cross resistance against fluconazole or other azoles [168,169,172], and cross resistance of *C. glabrata* of echinocandins and amphotericin B have also been reported [173]. Estimates regarding the percentage of non-susceptible or resistant *C. glabrata* clinical isolates against four antifungal drugs used in clinical practice, across several geographic regions are: fluconazole, 3.4%–70%; amphotericin B, 2.5%–60%; caspofungin, 1.3%–16.2%, and 5-flucytosine, 0.8%–35%, which are clearly concerning numbers [171,173,174,175,176,177,178,179,180,181,182,183,184,185,186,187,188,189,190,191,192,193]. Additionally, the latest cross-resistance between amphotericin B and azoles or caspofungin in *Candida* species are increasingly worrying [103,194,195,196,197]. Not in all cases, but in most of them, this multidrug resistance phenomenon depends on the activity of *ABC* transporters and *MFS* [198], which are known to be regulated by the Pdr1 transcription factor, which is recognized to be the major regulator of multidrug resistance in *C. glabrata* [94]. As previously explained, *C. glabrata* expresses three *ABC* transporters (*CDR1*, *CDR2*, and *SNQ2*). Studies revealed that the deletion of *CDR1* in an azole-resistant strain leads to the intracellular accumulation of fluconazole and hypersusceptibility to other azoles. The additional *CDR2* deletion worsens this phenotype [91,199]. Finally, the deletion of *SNQ2* leads to an amplified susceptibility to several azoles, but also to 4-nitroquinoline-*N*-oxide, in an azole-resistant strain [200]. Recently, Healey and colleagues [201] showed a mutator phenotype caused by a mismatch repair defect which is prevalent in *C. glabrata* clinical isolates. These strains possess alterations in mismatch repair gene *MSH2* which leads them to display an advanced predisposition to develop antifungal treatment in vitro and in mouse models of colonization. Also, the authors found that 55% of all *C. glabrata* that are recovered from patients have these genetic characteristics, which is clinically very concerning. The genetic mechanism involved in this process supports the acquisition of resistance to multiple antifungals, partially explaining the higher rates of triazole and multidrug resistance associated with *C. glabrata* [201]. Beforehand, Nishikawa and colleagues [202,203,204] identified an activator-targeted KIX domain in the human MED15 Mediator subunit that is structurally conserved in Gal11/Med15 Mediator subunits in fungi. This Gal11/Med15 KIX domain is involved in Pdr1 orthologues and in the clinically important *C. glabrata* multidrug resistance pathogenenis [205]. In a recent work, Nishikawa et al. [204] implemented a sequential biochemical and in vivo high-throughput screens to identify small-molecule inhibitors of the interaction of the *C. glabrata* Pdr1 activation domain with the *C. glabrata* Gal11A KIX domain, which is linked to the *C. glabrata* multidrug resistance. Results showed that iKIX1 inhibits Pdr1-dependent gene activation and re-sensitizes drug-resistant *C. glabrata* to azole antifungals in vitro and in animal models for disseminated and urinary tract *C. glabrata* infection [204]. The sirtuins Sir2 and Hst1 control the expression of several genes including adhesins required for host colonization and niacin transporters needed for growth in *C. glabrata*. With the knowledge that these sirtuins can be inactivated during infection, Orta-Zavalza et al. [206] proved that their inhibition could change the response of *C. glabrata* to other stressful conditions. The results showed that a deletion of *HST1* reduced the susceptibility of *C. glabrata* to fluconazole and hydrogen peroxide. Pdr1 and *CDR1* mediated the fluconazole resistance phenotype of the Δ *hst1* cells, while the transcriptional activator Msn4 and the catalase Cta1 were required to provide oxidative stress resistance. Also, the authors showed that the transcription factor Sum1 interacts with Hst1 and participates in the regulation of these genes. The findings state that *Hst1* acts as a regulator of stress resistance associated-genes [206].

*Candida* species seems to tolerate stress induced by weak acids, which appears to be a key factor in their persistence and virulence in antifungal drugs. *MFS* transporters are integrated into two families: the DHA1 (drug: H^+^ antiporter family 1), with 12 transmembrane domains, and the DHA2 (drug: H^+^ antiporter family 2), with 14 transmembrane domains. Other transporters, Qdr2 and Tpo3, have also been studied [198]. The first was acknowledged to be a cause of resistance to imidazoles (e.g., clotrimazole, miconazole, tioconazole, and ketoconazole) and was proven to play an active part in the efflux of these drugs. Its expression was found to be stimulated in clotrimazole-stressed cells, under Pdr1 control [207]. The second was demonstrated to be linked to the resistance to both imidazoles and triazoles (e.g., fluconazole), and to the polyamine spermine, found in high concentrations in the urogenital tract. The authors also found that *TPO3* was upregulated in *C. glabrata* cells exposed to spermine, in a Pdr1-dependent manner [198]. Likewise, Tpo3 appears to be linked to the efflux of azoles and spermine, and the control of the intracellular concentration of this polyamine appears to be important for azole resistance [208]. Another *MFS* H^+^ antiporter, Aqr1, from *C. glabrata*, was also identified by Costa et al. [209]. This *MFS* antiporter is a determinant of resistance to acetic acid, flucytosine, and clotrimazole (frequently found in the vaginal mucosa, probably contributing to the persistence in this niche). It is known that these antifungals act synergistically with acetic acid against this pathogen. Aqr1 (located in plasma membrane and in the membrane vesicles) was suggested to play a role in intermediating the extrusion of chemical compounds, dropping the intracellular accumulation of ^3^H-flucytosine and, in a minor degree, of ^3^H-clotrimazole, which is reliable with a direct role in antifungal drug efflux. When an *AQR1* deletion was performed, no effect could be noticed on the intracellular accumulation of 14C-acetic acid, thus suggesting that its role in acetic acid resistance may be indirect, possibly through the transport of a so far undisclosed physiological substrate. The pre-exposure to flucytosine or clotrimazole was found to make *C. glabrata* cells more tolerant to acetic acid stress. Therefore, Costa et al. [209] showed that Aqr1 is an antifungal drug resistance determinant and it may play an essential part in *C. glabrata* persistent colonization and multidrug resistance.

Hull et al. [210] identified a clinical isolate of *C. glabrata* (CG156) that displayed flocculent growth and cross-resistance to fluconazole, voriconazole, and amphotericin B. In this work, CG156 was found to be a low-efflux isolate and when grown on sterol-supplemented, its cultures reached higher cell densities, with shorter lag phases, and showed variations in cellular sterol composition that did not affect its azole-resistant phenotype. When this isolate was grown in the presence of ergosterol, it showed increased sensitivity to the polyene and when grown with cholesterol it became more resistant. The results therefore indicate that some clinical isolates might persist as slow-growing agents of chronic infections, possibly since they can survive without sterol auxotrophy; possess mutated Erg11; lack cellular ergosterol (high-level resistance to polyenes); and can opportunistically exploit a wide spectrum of host/environmental sterols for growth. The authors also indicate that the altered cellular sterol composition of CG156 may affect intracellular signalling and trafficking pathways, as the efflux machinery [199,211,212] and any transport proteins that are proposed to mediate azole import via facilitated diffusion [213].

Other cases of induced cross-resistance to azole drugs in *C. glabrata* were related to resistance against both azoles and amphotericin B [198,199,212] and related to prochloraz (an agricultural antifungal). The original mechanism responsible for this phenomenon was found to be the upregulation of multidrug transporters [214]. Also in *C. glabrata*, Flr1 has been proven to be involved in the resistance of benomyl (a pesticide used in agriculture), but no connection was found between this transporter and antifungal resistance [108].

Vermitsky and colleagues [94] showed that treatment with terbinafine (allylamines antifungal class), which targets the enzyme squalene epoxidase in the ergosterol biosynthetic pathway, presented upregulation on *ERG11* as previously reported, however, unlike the azoles, this drug had minimal effect on *CDR1* and *PDH1*. Several studies with histatins have been performed, since these salivary cationic proteins had been found to have effectiveness against diverse fungi [215,216,217,218,219]. Unfortunately, further studies disclosed that they have expressively less activity against *C. glabrata*, with current speculation suggesting that *C. glabrata* might escape histatin 5 activity by applying fermentative pathways, as theoretically glucose can either be fermented or assimilated. Moreover, *C. glabrata* is a Crabtree-positive fungus [220], thus its respiration could be negatively affected by certain levels of glucose [221]. The same results that confirmed an apparently fundamental and extensive resistance of *C. glabrata* to histatin 5 showed that it is not related to the resistance mechanisms of azoles.

## 5. Alternative Therapies for the Treatment of Infections Related to *Candida glabrata*

Considering the increasing number of *Candida* species with drug resistance, namely *C. glabrata*, the identification of efficient alternative therapies to the current antifungal agents is crucial. Many approaches are currently being pursued, including the development of novel antifungal agents, the exploitation of the antimicrobial properties of plant derivatives and honey, and the development of photodynamic therapy. An overview of the most recent advances in these approaches is provided in the following paragraphs.

### 5.1. Plant Essential Oils and Extracts

Despite the efforts to discover novel and more efficient chemical antifungal molecules, these are often associated with a variety of adverse side effects. This prompted the search for safer alternatives, among which are plants. Medicinal plants have been used since ancient times for therapeutic purposes. For example, superficial candidiasis is traditionally treated with a topical application of calendula and commiphora [222]. Currently, these plants are being investigated to determine the active principles responsible for their therapeutic effects and to understand how to apply these principles for antifungal treatment [223,224,225]. Several plant secondary metabolites such as tannins, terpenoids, alkaloids, flavonoids, and glycosides have been found to have antimicrobial properties in vitro [226,227]. Plant antifungal properties are less explored than antibacterial properties, but they are thought to derive from the same secondary metabolites. To explore the antifungal potential of plants, both essential oils and plant extracts have been used. Both are promising antifungal agents because of their relative safety, wide acceptance (a consequence of the traditional use), and are renewable in nature [228,229,230].

Plant essential oils are odorous volatile natural complex compounds. They are found only in 10% of the plant kingdom [231] and are generally in low quantities (rarely exceeding 1% of the plant mass) [232]. They are typically recovered from plants by distillation methods, are hydrophobic [233,234], and are composed of variable mixtures of many compounds (e.g., terpenoids such as alcohols, esters, aldehydes, ketones, ethers, phenols and epoxides, and low molecular weight aliphatic hydrocarbons [235,236]).

Likewise, plant extracts are of complex composition. These can be extracted from different plant organs, giving rise to a variable composition (in components and quantities) and therefore therapeutic effect. For example, *Mirtus communis* L. (myrtle) leaf and flower extracts are rich in tannins while the stem is rich in flavonoids [237]. Furthermore, the composition of the extracts is also variable according to the solvent used for extract preparation [238].

Several plant essential oils and extracts have been investigated for possible antifungal properties. These include those obtained from *Origanum vulgare* (oregano), *Cinnamomum zeylanicum* (cinnamon), *Lippia graveolens* (Mexican oregano), *Thymus vulgaris* (thyme), *Salvia officinalis* (sage), *Rosmarinus officinalis* (rosemary), *Ocimum basilicum* (basil), *Zingiber officinale* (ginger), *Eucalyptus globulus* (blue gum), *Juglans regia* (walnut), *Pterospartum tridentatum* (common poppy), *Rubus ulmifolius* (blackberry), and *Glycyrrhiza glabra* L. (licorice). Antimicrobial activity against *C. glabrata* isolates was observed for *Origanum vulgare*, *Lippia graveolens*, and *Cinnamomum zeylanicum* essential oils, with the first two being more active against fluconazole-susceptible *C. glabrata*, and the latter showing the best antifungal activity against the fluconazole-resistant *C. glabrata* isolates [239]. Extracts from *Juglans regia*, *Eucalyptus globulus*, *Pterospartum tridentatum*, and *Rubus ulmifolius* were also effective against several *Candida* species, especially *C. glabrata*, followed by *C. albicans*, *C. parapsilosis*, and *C. tropicalis*. The effects of *Juglans regia* and *Eucalyptus globulus* were assessed in more detail revealing a fungistatic and not fungicidal activity [240]. *Glycyrrhiza glabra* extracts have shown promising inhibitory results against several *Candida* strains, especially *C. tropicalis* and *C. glabrata*. These extracts have also demonstrated anti-biofilm activity against the two *Candida* species, although a double concentration of extract was generally required to obtain an antifungal effect in biofilm similar to that of planktonic cells [241].

In summary, plant essential oils and extracts are promising alternatives to current chemical antifungal agents. However, it is important to note that the establishment of standards for the therapeutic use of these plant derivatives to treat *Candida* species infections will require overcoming the variation in composition between samples, resulting from a multitude of factors that include, but are not restricted to, genotype, cultivation area, time of harvesting, and processing methods [242].

### 5.2. Honey

Honey is a natural sweet substance produced by honeybees from the nectar of flowers or from secretions of living parts of plants, which are transformed in the upper aero-digestive tract of the bee. Consequently, the chemical composition of honey varies depending on the botanical source. Honey is then stored in the honeycomb where it ripens and matures [243].

The first human use of honey traces back to 8000 years ago. Since then, honey has played an important role in traditional medicine, and is now also finding its place in modern medicine. In fact, studies have reported the bactericidal, bacteriostatic, antiviral, antioxidant, anti-inflammatory, and anti-tumoral properties of honey [244,245,246,247]. However, although a number of studies have investigated the antimicrobial properties of honey against bacteria, few have focused on its antifungal properties [248,249,250,251,252,253]. Nevertheless, the few reports currently available show promising results. Irish et al. [250] evaluated three floral honeys (Jarrah honey, Medihoney Antibacterial Honey Barrier, Comvita Would Care 18+) and one artificial honey simulating honey’s typical high sugar levels against *C. albicans*, *C. glabrata*, and *C. dubliniensis*. They found that *C. dubliniensis* was the most susceptible species to the activity of honey, while *C. glabrata* was the least susceptible. They also reported greater antifungal activities of the floral honeys compared to the artificial honey. This observation was also reported in the study of Estevinho et al. [243], where a synthetic honey solution was tested to determine the antifungal activity that was attributable to sugars, only to find that the activity was reduced compared to natural honey. It is therefore suggested that the component(s) of honey responsible for the antifungal properties are not sugar based.

Additional in vitro and in vivo evaluations are necessary to fully assess the antifungal potential of honey. For in vivo applications, honey may be limited to topical treatments, not being a viable option to treat candidaemia. However, honey may be used prophylactically to prevent more serious infections. A few studies have already demonstrated this possibility. For example, prevention of exit site infection by coating catheters with honey was found to be at least as effective as povidone iodine [254] or mupirocin [255].

### 5.3. Photodynamic Therapy (PDT)

Many studies have investigated the use of photodynamic therapy (PDT) to fight fungi infections [256]. PDT uses a photosensitive substance activated by a light source of a specific wavelength. The activation of photosensitizers added to cells and microorganisms, by an appropriate wavelength of light in the presence of oxygen, promote a phototoxic response of the cells, usually via oxidative damage [257]. The PDT sensitization depends on the parameters related to the concentration, time of incubation, and type of photosensitizer, as well as the physiological state of the microorganisms, time of exposure, and energy density of the laser [258,259]. Although photodynamic approaches are well established experimentally for the treatment of certain cutaneous infections, there is limited information about their mechanism of action for specific pathogens as well as the risks to healthy tissues [260].

The action of different photosensitizers, mainly phenothiazine dyes, porphyrins, and phthalocyanines, has been investigated. Many studies have demonstrated the efficacy of phenothiazine dyes such as methylene blue, new methylene blue, and toluidine blue in PDT for the reduction of fungi [256,258,259,261]. Malachite green, a cationic dye of the triarylmethane family (e.g., crystal violet) is another option for a photosensitizer. These dies have been shown to effectively reduce the number of *Candida* species cells. Dovigo et al. [262] observed that the fungicidal effect of PDT was strain-dependent. Significant decreases in biofilm viability were observed for three strains of *C. albicans* and for two strains of *C. glabrata*. Moreover, single-species biofilms were less susceptible to PDT than their planktonic counterparts.

As a consequence of the use of non-specific oxidizing agents, organisms resistant to conventional antifungal agents may be successfully killed by PDT and the development of resistance to this therapy seems unlikely, making this a very promising therapy [257]. In fact, the data to date suggests that photodynamic treatment approaches hold great promise for combating certain fungal pathogens.

### 5.4. Antifungal Agents with New Targets and New Sources

As a consequence of the increasing resistance demonstrated by *Candida* species to the currently available antifungal drugs, efforts are being made to develop novel and more effective antifungals. Different molecules are being discovered, synthesized, and evaluated for their capacity to control *Candida* species growth. For example, Vartak et al. [263] isolated a new polyene macrolide antibiotic from the fermentation broth of *Streptomyces* species. After purification, they evaluated the antimicrobial activity against several fungi (*Aspergillus fumigatus*, *C. albicans*, *C. krusei*, *C. glabrata*, *Cryptococcus neoformans*, *Trichophyton* species, and fluconazole-resistant *C. krusei* and *C. glabrata* strains) and Gram-positive and Gram-negative bacteria. They found the polyene macrolide to be specifically active against fungi, being unable to inhibit bacterial growth.

Glucosides modified in their saccharide units have been synthesized and their activity against *Candida* species evaluated by de Souza et al. [264]. One of the modified glucosides showed promising results, with fungistatic (threefold higher than fluconazole) and fungicidal activity against *C. glabrata*. The authors consider this compound to be a novel structural pattern in the development of new antifungal drugs. Different aldehydes, hydrazones, and hydrazines have also been assessed against several *Candida* species, among which 4-pyridin-2-ylbenzaldehyde and tert-butyl-(2*Z*)-2-(3,4,5-trihydroxybenzylidine)hydrazine carboxylate have shown the most promising results [265]. The activity of these compounds is thought to be on the fungal membrane. A series of fatty *N*-acyldiamines, prepared from fatty methyl esters and 1,2-ethylenediamine, 1,3-propanediamine, or 1,4-butanediamine, have also demonstrated moderate to good antifungal activity against *C. albicans*, *C. tropicalis*, *C. glabrata*, and *C. parapsilosis* [266].

Abrão et al. [267] evaluated the anti-candidal activity of new Mannich base-type eugenol derivatives and found that 4-allyl-2-methoxy-6-(morpholin-4-ylmethyl) phenyl benzoate and 4-{5-allyl-2[(4-chlorobenzoyl)oxy]-3-methoxybenzyl}morpholin-4-ium chloride were the most effective, particularly against *C. glabrata*, *C. albicans*, and *C. krusei*, with *IC*_50_ values below those of fluconazole. Raman et al. [268] evaluated the activity of amphiphilic, helical β-peptide structural mimetics of natural antimicrobial α-peptides against clinically isolated and drug resistant *Candida* strains. *Candida tropicalis* was the most susceptible to the activity of the β-peptide, while *C. glabrata* was the least susceptible. Interestingly, they report that β-peptides are mostly ineffective at disrupting *Candida* species biofilms, but they can prevent the formation of *C. albicans*, *C. glabrata*, *C. parapsilosis* and *C. tropicalis* biofilms. β-peptides thus seem to be a promising class of molecules to use as therapeutics.

Silva-Dias et al. [269] evaluated the antifungal properties of cerium, a lanthanide member, against planktonic and sessile *Candida* cells. The activity of cerium appears to result from severe cellular metabolic activity impairment and membrane damage. This compound was shown to effectively prevent biofilm formation both in vitro and in vivo in segments of polyurethane catheters in mouse models, and also to almost eradicate established biofilms when used at high concentrations. Cerium is therefore suggested as a possible antifungal agent to prevent the formation of biofilm-associated infections in clinical settings, for example, by catheter coating.

Additionally, antibacterial agents are being evaluated for their antifungal activity. For example, chloramphenicol, a bacteriostatic antibiotic, was evaluated against 30 representative yeast strains [270]. The antifungal activity of chloramphenicol was comparable to other known antifungal compounds (e.g., caspofungin acetate, ketoconazole, and metronidazole). However, it had no activity against most *C. albicans* tested, as well as *C. famata*, *C. glabrata*, *C. haemolonei* and *Cryptococcus neoformans*.

The combination of common antifungal agents with other antifungal molecules is also being assessed. For example, Ning et al. [271] has shown that the combination of epigallocatechin gallate (EGCG) with miconazole, fluconazole, or amphotericin B has a synergistic effect against planktonic and biofilm cells of *Candida* species (Fractional inhibitory concentrations (FIC) ≤ 50). They suggest that combined treatment of antifungal agents and EGCG may lower the dosages of antimycotics necessary to treat infections, thus preventing possible adverse effects and the emergence of resistant strains.

Another approach under consideration is the design of drugs with extended persistence and controlled release. For this purpose, nanotechnology creates new possibilities. For example, Perera et al. [272] evaluated the encapsulation of citric acid into a Mg-Al-layered double hydroxide (LDH). Citric acid has antifungal properties and its encapsulation would allow its slow release in topical skin formulations. The nanoparticles obtained were introduced into a body cream formulation, which demonstrated a prolonged slow release up to 8 h in aqueous medium under different pH values (3–5). The same body cream demonstrated an improved antifungal activity against *C. albicans* and *C. glabrata*, but not *C. tropicalis*. Also, Silva et al. [273] presented results with silver nanoparticles having a significant effect on reducing *C. glabrata* biofilm.

Defence mutualisms between social insects and microorganisms have been also largely studied, since the symbiotic nature of endophytic microorganisms favours metabolic interactions with their host and their environment, increasing the production of bioactive compounds [274]. Nirma et al. [275] reported the discovery of a *Pseudallescheria boydii* strain isolated from *Nasutitermes* species The microbial symbiont produces two metabolites with antifungal activity: tyroscherin and *N*-methyltyroscherin, shown to be effective antifungal agents with favourable selectivity indices for *C. albicans* and *C. parapsilosis*. Later, the same authors [276] discovered ilicicolinic acids A, C, and D and ilicicolinal isolated from a fungus (*Neonectria discophora* SNB-CN63) that was isolated from a termite (*Nasutitermes corniger)* nest in French Guiana, which showed in vitro against the same *Candida* species. Also in French Guiana, other authors [274] isolated fungi and bacteria from plants. Three active fungal extracts were fractionated, resulting in the isolation of eight compounds, which exhibited antifungal and cytotoxic potential against *C. albicans* ATCC10213.

## 6. Conclusions

Fungal infections have been increasing significantly in recent years, contributing to high morbidity and mortality, especially in immunocompromised individuals. The quick rise in the incidence of single, cross, and multidrug antifungal resistance within *C. glabrata* strains make it crucial to further increase the data on the virulence and resistance mechanisms associated with this species. Also, the capacity to form biofilms is one of the most important features in *Candida* species pathogenicity, creating a dangerous prospect of ineffective therapies against these infections. *Candida glabrata* biofilms are extremely refractory to antimicrobial therapy and more difficult to manage due to the natural properties mode of growth. They are able to withstand much higher concentrations of antifungal drugs compared with the planktonic cells, making *C. glabrata* biofilm infections extremely challenging to treat.

**Figure 2:**
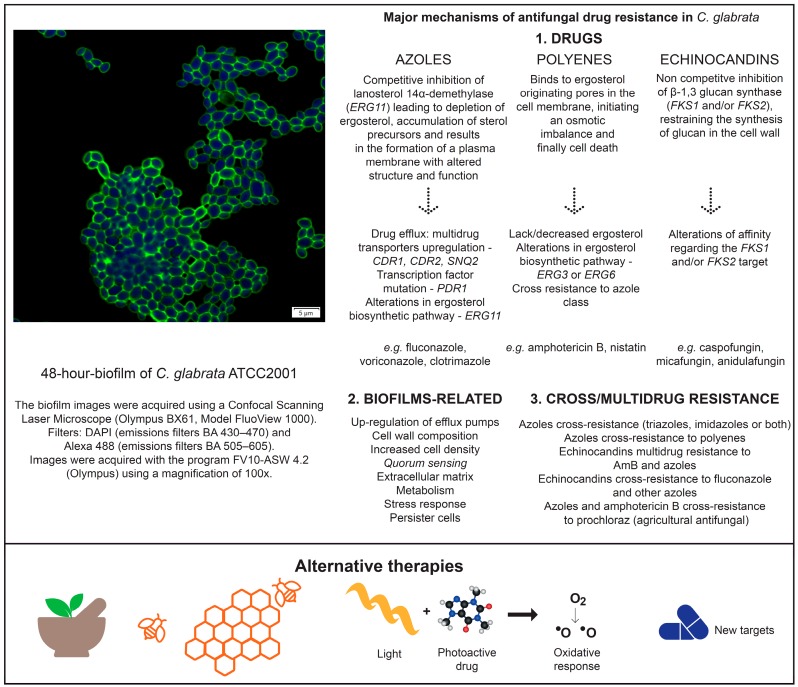
Mechanisms of antifungal resistance and alternatives therapies associated to *C. glabrata* biofilms.

The research for an improved comprehension on the mechanisms of drug resistance, but also the search for alternatives to antifungal therapies, is becoming more important over time. Figure 2 summarizes the mechanisms of antifungal resistance and the present alternative therapies related to *C. glabrata* biofilms.
